# Viability assessment and transplantation of fatty liver grafts using end‐ischemic normothermic machine perfusion

**DOI:** 10.1002/lt.26574

**Published:** 2022-10-10

**Authors:** Damiano Patrono, Riccardo De Carlis, Alessandro Gambella, Francesca Farnesi, Alice Podestà, Andrea Lauterio, Francesco Tandoi, Luciano De Carlis, Renato Romagnoli

**Affiliations:** 1 General Surgery 2U–Liver Transplant Unit, Azienda Ospedaliero Universitaria Città della Salute e della Scienza di Torino, University of Turin, Turin, Italy; 2 Department of General Surgery and Transplantation, Azienda Socio‐Sanitaria Territoriale Grande Ospedale Metropolitano Niguarda, Milan, Italy; 3 Pathology Unit, Department of Medical Sciences, University of Turin, Turin, Italy; 4 School of Medicine and Surgery, University of Milano‐Bicocca, Milan, Italy

## Abstract

End‐ischemic viability testing by normothermic machine perfusion (NMP) represents an effective strategy to recover liver grafts having initially been discarded for liver transplantation (LT). However, its results in the setting of significant (≥30%) macrovesicular steatosis (MaS) have not been specifically assessed. Prospectively maintained databases at two high‐volume LT centers in Northern Italy were searched to identify cases of end‐ischemic NMP performed to test the viability of livers with MaS ≥ 30% in the period from January 2019 to January 2022. A total of 14 cases were retrieved, representing 57.9% of NMP and 5.7% of all machine perfusion procedures. Of those patients, 10 (71%) received transplants. Two patients developed primary nonfunction (PNF) and required urgent re‐LT, and both were characterized by incomplete or suboptimal lactate clearance during NMP. PNF cases were also characterized by higher perfusate transaminases, lower hepatic artery and portal vein flows at 2 h, and a lack of glucose metabolism in one case. The remaining eight patients showed good liver function (Liver Graft Assessment Following Transplantation risk score, −1.9 [risk, 13.6%]; Early Allograft Failure Simplified Estimation score, −3.7 [risk, 2.6%]) and had a favorable postoperative course. Overall, NMP allowed successful transplantation of 57% of livers with moderate‐to‐severe MaS. Our findings suggest that prolonged observation (≥6 h) might be required for steatotic livers and that stable lactate clearance is a fundamental prerequisite for their use.

## INTRODUCTION

To expand the organ donor pool and improve access to liver transplantation (LT), the use of so‐called extended‐criteria donors has become commonplace in recent years. Frequently, extended‐criteria grafts are defined as those proceeding from donation after circulatory death (DCD), elderly donors, or livers with significant steatosis, in particular macrovesicular steatosis (MaS).^1^,^2^

The paradox of steatotic liver grafts is well known; despite apparent normal liver function in the donor, fatty livers are more susceptible to ischemia/reperfusion injury and are exposed to an increased risk of functioning poorly in the recipient, proportionally to the degree of MaS.^3^ Although the literature on the subject should be interpreted with caution because of the intra‐ and interobserver variabilities in steatosis assessments,^4^ the use of livers with moderate (≥30%) or severe (≥60%) MaS has been associated with an increased risk of graft dysfunction, primary nonfunction (PNF), acute kidney injury (AKI), and inferior 1‐year graft survival.^3^,^5^–^7^

The number of organ donors suffering from non–alcohol‐associated or metabolic dysfunction–associated liver disease^8^ is likely to increase in the future, as projections show that by 2030, the national prevalence of obesity (body mass index [BMI] ≥ 30) and severe obesity (BMI ≥ 35) will be 48.9% and 24.2%, respectively, in the United States.^9^ Accordingly, the prevalence of nonalcoholic fatty liver disease in the general population has been estimated to be 25.5%–29.2%,^10^ affecting also 10.6%–12.6% of patients with normal body weight.^11^ This is reflected in the rise of nonalcoholic steatohepatitis (NASH) and NASH‐related hepatocellular carcinoma (HCC) as indications for LT^12^ and has obvious implications also with regard to the organ donor pool. Thus, strategies allowing the safe use of steatotic grafts are urgently needed.

In the current scenario characterized by a growing prevalence of extended‐criteria donors, machine perfusion techniques have been introduced to improve organ preservation and overcome the limitations of static cold storage (SCS).^13^–^17^ Among them, normothermic machine perfusion (NMP), by mimicking a physiologic environment in which the liver is metabolically active, also allows the assessment of graft viability before transplantation into the recipient.^18^ Liver viability testing by using NMP, even if applied after an initial period of SCS (so‐called “back‐to‐base” approach),^18^–^24^ has been demonstrated to allow a safe expansion of the donor pool, enabling the successful transplantation of livers that would have otherwise been discarded.^18^ No study, however, has focused on the specific setting of steatotic liver grafts. Therefore, limited conclusions about the value of end‐ischemic NMP as applied to grafts with significant MaS can be drawn.

Prompted by the suboptimal results obtained with end‐ischemic hypothermic oxygenated machine perfusion for steatotic livers^25^–^29^ and pushed by the need to improve patient safety, NMP was introduced at our institutions for grafts with risk profiles that were deemed prohibitive, precluding their use without previous viability assessment. In most of these cases, MaS represented the main indication for NMP use.

Thus, with the aim of facilitating clinical decision making^30^ and refining criteria for liver viability assessment, this study sought to analyze the efficacy and safety of end‐ischemic NMP in the specific setting of moderate‐to‐severe MaS.

## MATERIALS AND METHODS

### Study design

Prospectively maintained databases of machine perfusion procedures and LTs performed between January 2019 and January 2022 were retrospectively analyzed to retrieve NMP procedures in liver grafts with MaS ≥ 30% performed with the aim of assessing graft viability before LT. The centers participating in the study were Azienda Ospedaliero Universitaria Città della Salute e della Scienza (Turin, Italy; *n* = 8) and Azienda Socio‐Sanitaria Territoriale Grande Ospedale Metropolitano Niguarda (Milan, Italy; *n* = 6). Analyzed data included baseline recipient and donor characteristics, NMP data, and post‐LT outcomes. The primary end point was utilization rate, defined as the percentage of transplanted livers among those evaluated with NMP. Secondary end points were patient and graft survival rates, rates of PNF and early allograft failure (EAF), and other measures of post‐LT outcomes. The study was conducted in accordance with the principles of the 2013 Declaration of Helsinki and was approved by institutional ethics committees.

### Liver procurement and normothermic machine perfusion

At procurement, the livers were cold flushed with Celsior solution (Institut Georges Lopez, Lissieu, France) and transported under SCS to the transplantation center. The indication for NMP was shared between the procuring and transplanting surgeons and was based on donor characteristics, the macroscopic aspect of the liver, and histological findings. Only grafts with histologically proven MaS ≥ 30% were included in this series. Graft histology was determined on liver biopsies obtained at procurement or before NMP, which were centrally reviewed by a single pathologist (A.G.) to homogenize MaS assessment.

After backtable preparation and cannulation, livers underwent a minimum of 4 h of NMP using either the OrganOx Metra (OrganOx, Oxford, UK) or LiverAssist (XVivo, Groningen, The Netherlands) devices. Reconstruction of aberrant hepatic arteries, if any, was performed during backtable preparation, before connecting the liver to the NMP device. A total of 20–30 mEq of sodium bicarbonate was added to the perfusate to equilibrate pH during the priming phase. Perfusate composition and machine perfusion protocols for each device have been described elsewhere.^15^,^31^ During NMP, perfusate samples were collected 15, 30, 60, 90, and 120 minutes after NMP start and then hourly to determine blood gas, lactate, glucose, and electrolyte levels. Perfusate aspartate aminotransferase (AST), alanine aminotransferase (ALT), and lactate dehydrogenase (LDH) levels were measured at 2 and 4 h of NMP. The primary criterion for livers to be considered for transplantation was lactate level ≤4 mmoL/L at 2 h of NMP and at least three of the following criteria: stable pH ≥7.3 without the need for repeated bicarbonate supplementation, evidence of glucose metabolism, bile production ≥2 ml/h, macroscopically homogeneous perfusion, hepatic artery (HA) flow ≥ 150 ml/min, and portal vein (PV) flow ≥ 500 ml/min. Bile samples were not systematically analyzed.

Livers that were deemed suitable for LT were preferentially allocated to recipients with low Model for End‐Stage Liver Disease (MELD) scores while considering donor–recipient size matching. LT was performed using the piggyback technique and portal reperfusion first. Standard immunosuppression included induction with basiliximab, tacrolimus, steroids, and mycophenolate mofetil.

### Definitions

Postreperfusion syndrome was defined according to Aggarwal et al.^32^ and Hilmi et al.^33^ Postreperfusion syndrome was defined as severe when associated with severe hemodynamic instability, persistent hypotension (more than 30% of the anhepatic level), asystole, or hemodynamically significant arrhythmias.^33^ PNF was defined as severe graft dysfunction determining patient death or requiring re‐LT within 7 days of LT in the absence of technical or immunological causes.^34^ EAF was defined as listing for re‐LT or patient death for any cause within 90 days of transplant.^35^ Other outcome measures, including early allograft dysfunction (EAD),^36^ AKI,^37^ Liver Graft Assessment Following Transplantation (L‐GrAFT) score,^38^ Early Allograft Failure Simplified Estimation (EASE) score,^35^ and Comprehensive Complication Index (CCI)^39^ score, were defined according to the original publications.

### Statistical analysis

Variables were reported as median (interquartile range [IQR]) or count (percentage), as appropriate. Mann‐Whitney U, Fisher's exact, and chi‐square tests were used to compare variables among different groups. Statistical analysis and data visualization were performed using R Version 4.1.1 (R Foundation for Statistical Computing, Vienna, Austria).

## RESULTS

**FIGURE 1:**
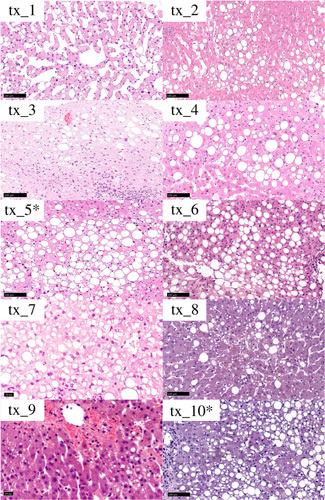
Histology of transplanted livers before NMP. Livers marked with an asterisk were those developing PNF. All cases presented with large‐droplet steatosis, whereas the tx_1 case was also characterized by a scattered sinusoidal dilation.

**FIGURE 2:**
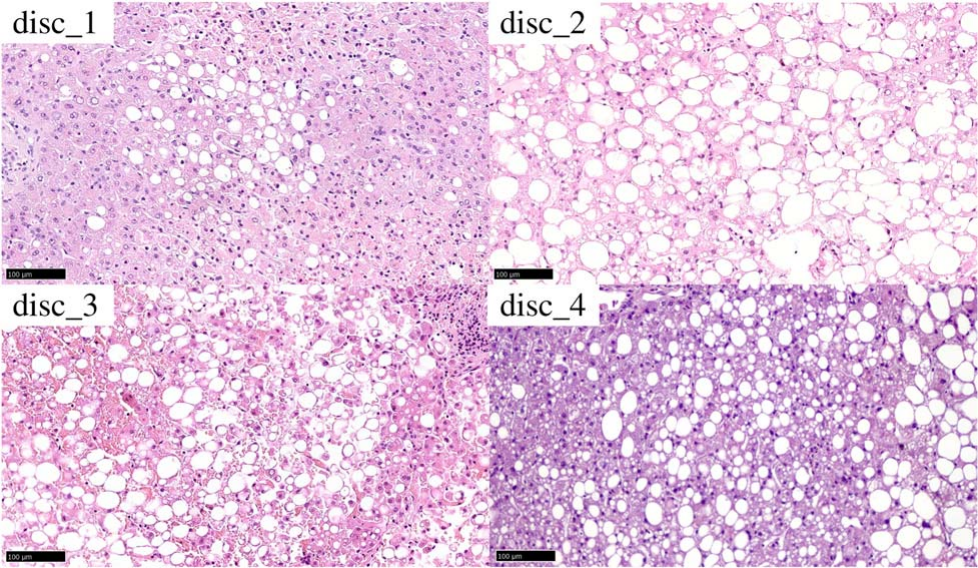
Histology of discarded livers before NMP

During the study period, 25 grafts were evaluated by NMP. In 14 cases (56%), the indication for viability assessment was based on graft steatosis, representing the cohort included in this study (Table 1). Of the 14 included liver grafts, 10 were transplanted (Figure 1) and four were discarded (Figure 2). The 10 (73%) transplanted grafts represented 1.3% of all LTs, 5.7% of LTs performed using any machine perfusion technique, and 57.9% of LTs performed using NMP during the study period.

**TABLE 1:** Synoptic view of study cohort

Identification no.	Donor type	Cause of death	Donor age (years)	Donor BMI	Liver weight (g)	Macrosteatosis (%)	Cold ischemia time (min)	Lactate clearance	Stable pH	Homogeneous perfusion	Artery flow >150 ml/min	PV flow >500 ml/min	Glucose metabolism	Bile production	Outcome
tx_1	DBD	Cerebrovascular	79	35	1940	30	461	✓	✓	✓	✓	✓	✓	✓	Functioning
tx_2	DCD^a^	Cerebrovascular	45	38	2140	35	248	✓	✓	✓	✓	✓	✓	✓	Functioning
tx_3	DBD	Cerebrovascular	59	35	2700	40	315	✓	✓	✓	✓	✓	✓	✓	Functioning
tx_4	DBD	Cerebrovascular	74	36	2600	30	241	✓	✓	✓	✓	✓	✓	✓	Functioning
**tx_5**	**DBD**	**Anoxic brain injury**	**72**	**53**	**2450**	**30**	**241**	**⨯**	**✓**	**✓**	**✓**	**✓**	**✓**	**✓**	**PNF**
tx_6	DBD	Trauma	50	25	2233	50	330	✓	✓	✓	✓	✓	✓	✓	Functioning
tx_7	DBD	Anoxic brain injury	51	36	2381	60	103	✓	✓	✓	✓	✓	✓	✓	Functioning
tx_8	DBD	Anoxic brain injury	49	34	2139	30	198	✓	✓	✓	✓	✓	✓	✓	Functioning
tx_9	DBD	Cerebrovascular	63	22	1844	40	240	✓	✓	✓	✓	✓	✓	✓	Functioning
tx_**10**	**DBD**	**Cerebrovascular**	**68**	**27**	**1690**	**50**	**465**	**⨯**	**✓**	**✓**	**✓**	**✓**	**⨯**	**✓**	**PNF**
disc_1	DBD	Cerebrovascular	79	37	2450	30	549	⨯	⨯	⨯	✓	✓	⨯	⨯	Discarded
disc_2	DBD	Cerebrovascular	48	35	2780	90	300	⨯	⨯	⨯	✓	✓	⨯	⨯	Discarded
disc_3	DBD	Cerebrovascular	64	34	3150	80	315	⨯	⨯	⨯	⨯	✓	⨯	✓	Discarded
disc_4	DBD	Cerebrovascular	68	34	2500	45	360	⨯	⨯	⨯	✓	✓	⨯	⨯	Discarded

*Note*: Lines in bold indicate livers that were transplanted and developed primary non‐function.

Abbreviations: BMI, body mass index; DBD, donation after brain death; DCD, donation after circulatory death; PNF, primary nonfunction; PV, portal vein.

a Maastricht category 3 DCD donor. The liver was retrieved after a functional warm ischemia time of 65 minutes followed by 227 minutes of normothermic regional perfusion.

Baseline donor and graft characteristics as well as the results of viability assessments and graft outcomes are presented in Table 1. All but one liver proceeded from donation after brain death (DBD) donors. The only Maastricht Category 3 DCD liver had 65 minutes of functional warm ischemia time and was procured after 227 minutes of normothermic regional perfusion, during which it met all viability criteria.^40^,^41^ However, given the marked steatosis, further assessment by NMP was deemed indicated. Median (IQR) donor age and BMI were 61 years (49, 71 years) and 35 (28, 36), respectively. Liver weight and MaS percentage, as initially assessed by the on‐call pathologist, were 2307 g (1990, 2562 g) and 40% (30%, 57%), respectively. NMP was initiated after a median cold ischemia time of 307 minutes (241, 349 minutes) and continued for 323 minutes (296, 360 minutes).

Livers were preferentially allocated to patients with low MELD (14 [9, 16]) and MELD‐sodium (14 [9, 17]) scores, of whom nine (82%) underwent LT for HCC. Median recipient BMI was 27 (26, 29), reflecting the attention to size matching during the allocation process.

### Viability assessment of discarded livers

**FIGURE 3:**
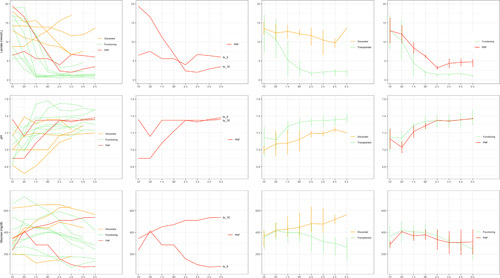
Perfusate parameters during NMP according to use and outcome. In the third and fourth columns, the lines and vertical error bars represent medians and IQRs at different time points during NMP. Abbreviations: ', minutes; h, hours.

**FIGURE 4:**
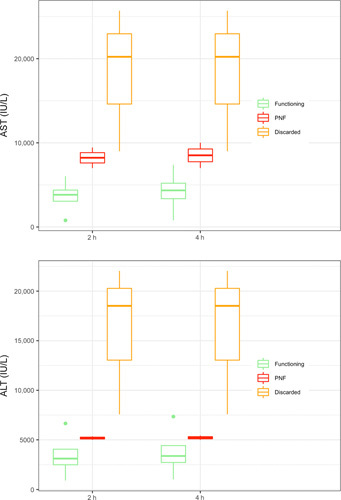
The 2‐h and 4‐h perfusate transaminases according to liver outcome

Donor and graft characteristics were comparable between transplanted and discarded grafts (Table 2), whereas perfusate and NMP parameters were different, as discarded livers showed higher levels of perfusate lactate, potassium, AST, ALT, and LDH; lower flow into the HA and PV; lower perfusate pH levels despite repeated bicarbonate administrations; and no evidence of glucose metabolism (Figures 3 and 4, Table 3).

**TABLE 2:** Donor features according to the result of the viability assessment and outcome after transplant

Donor characteristics and graft histology	Discarded	Transplanted	*p* value	Functioning	PNF	*p* value
n	4	10		8	2	
Donor type			1.00			1.00
DBD	4 (100)	9 (90)		7 (88)	2 (100)	
Category 3 DCD	0 (0)	1 (10)		1 (12)	0 (0)	
Age, years	66 (60, 71)	61 (50, 71)	0.62	55 (50, 66)	70 (69, 71)	0.30
BMI	35 (34, 35)	35 (28, 36)	1.00	35 (32, 36)	40 (33, 47)	0.60
ITU stay, days	1.5 (1.0, 3.0)	3.0 (2.0, 5.8)	0.20	2.5 (2.0, 5.8)	4.5 (3.8, 5.2)	0.51
Sodium, mmol/L	147 (146, 150)	146 (144, 157)	0.78	147 (145, 160)	144 (144, 145)	0.36
AST, IU/L	197 (98, 616)	74 (33, 113)	0.18	88 (58, 139)	36 (33, 38)	0.30
ALT, IU/L	141 (57, 443)	48 (30, 121)	0.16	82 (34, 125)	33 (30, 37)	0.30
GGT, IU/L	88 (42, 142)	74 (35, 146)	0.72	74 (33, 132)	151 (95, 206)	0.60
Creatinine, mg/dl	1.5 (1.3, 2.0)	1.7 (0.9, 2.5)	0.83	2.2 (1.0, 2.7)	1.0 (0.8, 1.2)	0.30
Cold ischemia time, min	337 (311, 407)	244 (240, 326)	0.14	244 (229, 319)	353 (297, 409)	0.36
NMP time, min	284 (250, 306.5)	332 (300, 374)	0.16	302 (285, 365)	425 (392, 457)	0.15
Macrosteatosis, %	47 (41, 52)	40 (31, 57)	0.83	40 (34, 52)	55 (42, 67)	1.00
Microsteatosis, %	10 (5, 25)	20 (2, 27)	0.86	20 (7, 30)	10 (5, 15)	0.42

*Note*: Data are presented as count (percentage) or median (IQR).

Abbreviations: ALT, alanine aminotransferase; AST, aspartate aminotransferase; BMI, body mass index; DBD, donation after brain death; DCD, donation after circulatory death; GGT, gamma‐glutamyltransferase; IQR, interquartile range; ITU, intensive therapy unit; NMP, normothermic machine perfusion; PNF, primary nonfunction.

**TABLE 3:** NMP parameters according to the result of the viability assessment and outcome after transplant

	Discarded	Transplanted	*p* value^a^	Functioning	PNF	*p* value^b^	*p* value^c^
n	4	10		8	2		
Second‐hour NMP parameters							
Lactate, mmol/L	11.1 (9.1, 13.7)	1.4 (1.1, 2.2)	<0.01	1.3 (1.0, 1.5)	3.2 (2.8, 3.6)	0.07	0.01
pH	7.2 (7.1, 7.2)	7.3 (7.3, 7.4)	0.07	7.3 (7.3, 7.4)	7.3 (7.3, 7.3)	0.60	0.06
Potassium, mmol/L	19.0 (15.3, 19.2)	5.4 (3.6, 7.8)	0.07	5.1 (3.2, 6.9)	7.4 (6.2, 8.5)	0.30	0.06
Glucose, mg/dl	561 (407, 635)	380 (225, 482)	0.26	380 (325, 427)	334 (245, 422)	1.00	0.23
HA flow, ml/min/kg	101 (91, 122)	217 (168, 234)	0.01	224 (204, 257)	140 (129, 152)	0.04	0.01
PV flow, ml/min/kg	358 (319, 409)	500 (468, 567)	0.01	514 (496, 640)	431 (423, 440)	0.04	0.01
Bile production, ml	2.5 (0.0, 6.2)	8.0 (5.0, 27.5)	0.07	8.0 (5.0, 22.5)	47.5 (26.2, 68.8)	0.69	0.08
AST, IU/L	20,225 (14,615, 22,960)	4931 (3817, 6756)	0.04	3823 (3053, 4383)	8222 (7611, 8833)	0.06	0.03
ALT, IU/L	18,510 (13,045, 20,270)	4099 (3071, 5271)	0.02	3113 (2496, 4064)	5181 (5090, 5271)	0.35	0.03
Fourth‐hour NMP parameters							
Lactate, mmol/L	9.7 (8.6, 10.8)	1.4 (1.1, 2.7)	0.03	1.3 (1.1, 1.6)	4.6 (3.8, 5.5)	0.07	0.04
pH	7.2 (7.2, 7.3)	7.4 (7.3, 7.4)	0.13	7.3 (7.3, 7.4)	7.4 (7.4, 7.4)	0.90	0.19
Potassium, mmol/L	18.7 (18.5, 18.9)	4.3 (3.2, 7.8)	0.03	3.6 (2.9, 6.4)	6.7 (5.8, 7.6)	0.43	0.04
Glucose, mg/dl	519 (480, 557)	311 (191, 352)	0.09	311 (230, 341)	308 (195, 422)	1.00	0.07
HA flow, ml/min/kg	159 (151, 222)	173 (161, 221)	0.84	198 (171, 230)	140 (129, 152)	0.10	0.61
PV flow, ml/min/kg	396 (341, 422)	548 (501, 633)	0.02	548 (514, 712)	520 (485, 556)	0.50	0.02
Bile production, ml	2.5 (0.0, 13.8)	80 (15.2, 115.0)	0.02	80 (14.5, 105.0)	75.5 (45.2, 105.8)	0.79	0.03
AST, IU/L	20,225 (14,615, 22,960)	5738 (4286, 7287)	0.04	4350 (3362, 5203)	8512 (7756, 9268)	0.16	0.03
ALT, IU/L	18,510 (13,045, 20,270)	4232 (3335, 5324)	0.02	3378 (2725, 4435)	5216 (5108, 5325)	0.35	0.03
Other NMP parameters							
NaHCO_3_ added, mEq	75.0 (58.8, 87.5)	12.5 (2.5, 37.5)	0.02	12.5 (7.5, 32.5)	20.0 (10.0, 30.0)	0.89	0.03
Cumulative bile production, ml	2.5 (0.0, 18.8)	95 (41.2, 187.5)	0.02	95 (43.8, 167.5)	115 (72.5, 157.5)	0.79	0.03

*Note*: Data are presented as counts (percentage) or median (IQR).

Abbreviations: ALT, alanine aminotransferase; AST, aspartate aminotransferase; HA, hepatic artery; IQR, interquartile range; NMP, normothermic machine perfusion; PNF, primary nonfunction; PV, portal vein.

a *p* values refer to Mann–Whitney U test results comparing discarded versus transplanted livers.

b *p* values refer to Mann–Whitney U test results comparing functioning versus PNF livers.

c *p* values refer to Mann–Whitney U test results comparing discarded versus functioning livers.

### Viability assessment and outcome of transplanted livers

Patient outcomes are summarized in Table 4. Two patients (one at each participating center) developed PNF, characterized by severe postreperfusion syndrome, refractory acidosis, hemodynamic instability, and severe AKI requiring renal replacement therapy. Recipients developing PNF showed high AST peak (tx_5, 10,187 IU/L; tx_10, >7000 IU/L), whereas the ALT peak was lower (tx_5, 2051 IU/L; tx_10, 287 IU/L). Both patients required urgent re‐LT 2 and 3 days after initial LT, respectively.

**TABLE 4:** Outcome

*n*		14
Utilization rate	Discarded	4 (29)
	Transplanted	10 (71)
Outcome	Discarded	4 (29)
	EAF	2 (14)
	Functioning	8 (57)
Outcomes in patients who received transplants		
*n*		10
AST peak, IU/L		2292 (983, 6330)
ALT peak, IU/L		697 (402, 1831)
EAD		5 (50)
L‐GrAFT score^a^		−1.9 (−2.4, −1.0)
L‐GrAFT score, risk %^a^		13.6 (8.5, 26.7)
EASE score^a^		−3.7 (−4.0, −2.8)
EASE score, risk %^a^		2.6 (2.0, 6.0)
AKI stage	0	4 (40)
	1	2 (20)
	2	1 (10)
	3	3 (30)
Dialysis after LT		2 (20)
Clavien‐Dindo classification ≥3b		4 (40)
CCI at discharge		35.5 (29.9, 38.8)
ICU stay, days		5.0 (2.2, 10.8)
Hospital stay, days		16.5 (13.2, 24.2)

*Note*: Data are presented as counts (percentage) or median (IQR).

Abbreviations: AKI, acute kidney injury; ALT, alanine aminotransferase; AST, aspartate aminotransferase; CCI, Comprehensive Complication Index; EAD, early allograft dysfunction; EAF, early allograft failure; EASE, Early Allograft Failure Simplified Estimation; ICU, intensive care unit; IQR, interquartile range; L‐GrAFT, Liver Graft Assessment Following Transplantation; LT, liver transplantation.

^a^ L‐GrAFT and EASE scores could be calculated only in patients not suffering from EAF.

The first PNF liver (tx_5) was characterized by insufficient lactate clearance during NMP (Figure 3). At 2 h of perfusion, perfusate lactate was 4 mmol/L, and all other viability criteria, including evidence of glucose metabolism, were met. Of note, the liver was producing bile (20 ml at 2 h), and bile composition, which was available for this case, was as follows: pH, 7.69; HCO_3_−, 37.2 mmol/L; glucose, <4 mg/dl; difference between perfusate and bile glucose, 153 mg/dl. Despite some concerns as a result of the slow lactate clearance, the liver was deemed transplantable, and the LT operation was started. Unfortunately, although all other parameters were stable, the lactate level increased to 6.7 mmol/L at 3 h and only slightly decreased to 6 mmol/L at 5 h. Second‐hour perfusate AST and ALT levels became available and were 9444 IU/L and 4433 IU/L, respectively. The 4‐h perfusate AST and ALT levels were only slightly increased (AST, 10,024 IU/L; ALT, 5433 IU/L). As donor hepatectomy was already at an advanced state, the procedure could not be aborted, and the liver was transplanted.

The second PNF liver (tx_10) initially showed good lactate clearance (second and third hour levels = 2.35 and 2.02 mmol/L, respectively) followed by a rebound in lactate levels up to 3.5 mmol/L at 5 h of NMP. This liver also failed to metabolize glucose, whereas other viability parameters (pH maintenance, bile production, macroscopic aspect, and vascular flows) were met. Perfusate AST and ALT levels at 2 and 4 h of NMP became available only after NMP had ended and were constantly above the laboratory determination limit (7000 IU/L for AST and 5000 IU/L for ALT). Bile produced during NMP (5 ml during the first 2 h and 30 ml at 5 h) was not analyzed in this case.

Overall, the common feature of livers developing PNF was insufficient or suboptimal lactate clearance, which was associated with high perfusate transaminases levels and lack of glucose metabolism in one case (Table 3, Figures 3 and 4). HA and PV flows were also lower in PNF livers at 2 h of NMP, whereas flows were comparable at 4 h of perfusion. It should be noted, however, that one liver in this series that also showed insufficient lactate clearance (Figure 3) subsequently had excellent function after LT. Furthermore, high perfusate transaminase levels were also observed in livers that subsequently showed good function (Figure 4, Table 3).

Regarding the histopathology of the two PNF cases, the tx_5 liver presented large‐droplet steatosis only, showing a unique large lipid vacuole per hepatocyte leading to an overall increased cellular size compared with nearby hepatocytes and displacing the nucleus to the periphery of the cell. Steatosis presented a zonal pattern of distribution, and within the areas of steatosis, hepatocytes presented features of collapse/compression (cell and cytoplasm shrinkage). There was a mild inflammatory infiltrate mainly represented by lymphocytes and neutrophils located in the lobules. No inflammation or major signs of injury were observed along the portal tracts. The tx_10 liver presented similar histopathological features but with combined small‐ and large‐droplet steatosis. Similar to tx_5, collapsing hepatocytes were noticed in steatotic areas, whereas the inflammatory infiltrate was more conspicuous, and the relative neutrophils component was more prevalent. In addition, portal tracts showed mild lymphocytes infiltrate but with no features of specific immune‐mediated injury.

### Comparison between discarded and transplanted viable livers

After the exclusion of the two PNF cases, NMP parameters were compared between the discarded and transplanted viable livers and showed lower perfusate lactate, potassium, and transaminase levels in viable livers at 2 and 4 h of NMP (Table 3). Viable livers were also characterized by higher cumulative bile production and a lower requirement for sodium bicarbonate during NMP. Differences in perfusate glucose levels became more evident after 4 h of NMP. HA and PV flows were significantly higher in viable livers at 2 h of NMP. At 4 h, the PV flow remained higher, whereas the HA flow was comparable.

Overall, utilization rate was 71%, whereas successful utilization rate, that is, the proportion of grafts transplanted and not suffering from EAF, was 57%. The recipients of grafts not suffering from EAF had a favorable postoperative course, as reflected by the low L‐GrAFT (−1.9; risk, 13.6%), EASE (−3.7; risk, 2.6%), and CCI (35.5) scores (Table 4). After a median follow‐up of 12 months (7, 26.5 months), the patient and graft survival rates are 100% and 80%, and no case of ischemic cholangiopathy has been observed. Besides the two PNF cases, no other patient required re‐LT during follow‐up.

## DISCUSSION

This study shows that, by using end‐ischemic NMP, approximately 60% of liver grafts with moderate‐to‐severe MaS can be transplanted with good outcomes. Although this compares negatively with previously published studies,^18^,^20^,^21^,^23^ which showed a recovery rate of about 70%, we should stress the exceptional nature of the cases included in this series. Indeed, these were highly selected livers evaluated at two high‐volume centers with broad experience with extended‐criteria donors that share the same philosophy toward machine perfusion, that is, prevalently using hypothermic oxygenated machine perfusion to reduce ischemia/reperfusion injury^26^,^42^,^43^ and reserving NMP to very high‐risk cases. Furthermore, although MaS ≥ 30% was the common characteristic of included cases, additional risk factors, including elevated donor age, were frequently present. Thus, the fact that more than half of these livers were transplanted and showed good function postoperatively appears to be a positive finding.

The downside of the reported experience is represented by the two PNF cases in our series, which points to the difficulty of assessing viability of these high‐risk livers. The main feature of the PNF cases was insufficient or suboptimal lactate clearance during NMP, which highlights the importance of this parameter in defining liver viability. Lactate perfusate levels during NMP reflect the balance between production and clearance. As the liver has a broadly redundant capacity to metabolize lactate,^44^,^45^ failure to metabolize lactate during NMP is generally considered as a sign of severe hepatocellular injury. In the closed NMP circuit, lactate production derives from erythrocyte metabolism, which is exclusively anaerobic, or from hypoperfused or necrotic areas of liver parenchyma. Incomplete clearance or a rebound in perfusate lactate levels might be indicative of an unbalance between production and metabolism and may suggest poor residual liver function. The choice of 4 mmol/L at 2 h of NMP as a threshold for lactate clearance in our series, which is higher than what has been proposed by most authors,^18^,^21^,^24^,^46^–^48^ was based on several considerations. First, a similar lactate threshold, although for a longer observation time, was adopted by the Cleveland Clinic group with optimal results.^23^ Second, Hann et al. from the Birmingham group^49^ suggested that slow lactate clearance might not represent an absolute contraindication to the use of DBD livers. Third, in our experience, suboptimal lactate clearance has not been invariably associated with poor outcomes. As an example, one liver in this series that did not completely clear lactate exhibited good function after LT, and the recipient had a favorable postoperative course (Figure 3). In retrospect, we acknowledge that the tx_5 liver should not have been transplanted, and we learned from this case that a longer evaluation time, ideally ≥6 h, is warranted to adequately evaluate steatotic grafts. The second case is undoubtedly more problematic, as the warning signs were more subtle in this case. Although it can be argued that the PNF cases were also characterized by higher perfusate transaminases levels and lower HA and PV flows at 2 h of NMP and a lack of glucose metabolism in the second case, our experience reflects the difficulty of viability assessment in borderline cases. As suggested by the recent series from the Groningen^48^ and Cambridge^50^ groups, viability assessment during NMP is still an evolving concept, and at present, given the lack of strong evidence, it would be difficult to formulate strong recommendations about what criteria should be unavoidably fulfilled and the degree of deviance that might be accepted. Putting together all of the pieces of the puzzle while appropriately weighing their importance might be particularly arduous, especially when dealing with particularly high‐risk livers. This highlights the urgent need for new markers of injury and function to be added to the armamentarium of currently available viability criteria.^51^,^52^

Besides refining viability criteria, which applies to all livers subjected to NMP, the real challenge with steatotic livers appears to be finding a strategy to increase their use rate. As proposed by the Groningen group,^48^ a possible approach could be a short period of hypothermic oxygenated perfusion to resuscitate mitochondria followed by controlled oxygenated rewarming and subsequent viability testing using NMP. However, given the extreme susceptibility of steatotic livers to the damage induced by SCS, any end‐ischemic approach could still be suboptimal. Although more logistically demanding, upfront NMP^14^,^15^ initiated at the donor hospital could have a prominent role in this setting, as it would significantly reduce initial cold ischemia time and possibly increase the percentage of viable livers to be transplanted. Pushing this concept even further, ischemia‐free LT,^53^ by which ischemia is completely avoided, could represent another interesting option.

To define which approach could increase the safe use of steatotic livers, the prerequisite for future randomized studies will be a uniform and consistent assessment of MaS, allowing a comparison of data across different studies. Steatosis represents probably the most concerning histopathological feature with regard to its impact on LT outcomes. However, as recently pointed out by the Banff guidelines,^54^ there are currently no validated cutoffs to predict liver graft survival or PNF based on liver biopsy assessment. Our PNF cases both presented a relatively high percentages of steatosis, most of which was represented by large‐droplet steatosis, which is specifically related to an increased risk of poor function after transplantation. Also, areas of large‐droplet steatosis were associated with the presence of collapsing hepatocytes, whereas inflammatory infiltrate was present, but not severe. To overcome the limitations attributed to interobserver variability in steatosis assessment and to allow a correct interpretation of our data, we chose to present histological features of the livers included in this series. In the future, wider implementation of the 2019 Banff consensus recommendations for liver steatosis definition and grading^54^ will hopefully increase the comparability across different studies.

Limitations of our study include the low number of cases and the lack of evaluation of cholangiocellular viability biomarkers.^55^ The importance of bile composition to assess cholangiocyte viability and predict the development of ischemic cholangiopathy progressively emerged throughout the study period. As bile samples were not systematically analyzed during this study, these data could not be presented. However, as the main issue with steatotic livers is postoperative function, the choice to focus on markers of hepatocellular function could be justified.

In conclusion, in our experience, end‐ischemic NMP allowed the successful transplantation of about 60% of livers with moderate‐to‐severe MaS. Viability assessment of these livers, in particular effective and stable lactate clearance, must be very rigorous, as PNF is still possible despite most other viability criteria being met, highlighting the need for new reliable viability biomarkers. Further studies are needed to investigate whether alternative approaches, such as preconditioning by hypothermic oxygenated machine perfusion before end‐ischemic NMP, upfront NMP, or ischemia‐free LT, may increase the use rate and improve the outcomes of these high‐risk livers.
